# Subtypes of tail spike proteins predicts the host range of *Ackermannviridae* phages

**DOI:** 10.1016/j.csbj.2021.08.030

**Published:** 2021-08-21

**Authors:** Anders Nørgaard Sørensen, Cedric Woudstra, Martine C. Holst Sørensen, Lone Brøndsted

**Affiliations:** Department of Veterinary and Animal Sciences, University of Copenhagen, Stigbøjlen 4, 1870 Frederiksberg C, Denmark

**Keywords:** ANI, Average nucleotide identity, CPS, Capsular polysaccharide, EOP, Efficiency of plating, LB, Luria-Bertani, LPS, Lipopolysaccharide, NCBI, National Center for Biotechnology Information, ORF, Open reading frame, PFU, Plaque formation unit, RBP, Receptor binding protein, TSP, Tail spike protein, VriC, Virulence-associated protein, Bacteriophage, *Ackermannviridae* family, Receptor-binding proteins, Tail spike proteins, Host range, O-antigen, *Escherichia coli* O:157, *Salmonella*

## Abstract

Phages belonging to the *Ackermannviridae* family encode up to four tail spike proteins (TSPs), each recognizing a specific receptor of their bacterial hosts. Here, we determined the TSPs diversity of 99 *Ackermannviridae* phages by performing a comprehensive *in silico* analysis. Based on sequence diversity, we assigned all TSPs into distinctive subtypes of TSP1, TSP2, TSP3 and TSP4, and found each TSP subtype to be specifically associated with the genera (*Kuttervirus*, *Agtrevirus*, *Limestonevirus, Taipeivirus*) of the *Ackermannviridae* family. Further analysis showed that the N-terminal XD1 and XD2 domains in TSP2 and TSP4, hinging the four TSPs together, are preserved. In contrast, the C-terminal receptor binding modules were only conserved within TSP subtypes, except for some *Kuttervirus* TSP1s and TSP3s that were similar to specific TSP4s. A conserved motif in TSP1, TSP3 and TSP4 of *Kuttervirus* phages may allow recombination between receptor binding modules, thus altering host recognition. The receptors for numerous uncharacterized phages expressing TSPs in the same subtypes were predicted using previous host range data. To validate our predictions, we experimentally determined the host recognition of three of the four TSPs expressed by kuttervirus S117. We confirmed that S117 TSP1 and TSP2 bind to their predicted host receptors, and identified the receptor for TSP3, which is shared by 51 other *Kuttervirus* phages. *Kuttervirus* phages were thus shown encode a vast genetic diversity of potentially exchangeable TSPs influencing host recognition. Overall, our study demonstrates that comprehensive *in silico* and host range analysis of TSPs can predict host recognition of *Ackermannviridae* phages.

## Highlights

• TSP subtypes are specifically associated with the phage genera.
• Receptor binding modules can be swapped among TSPs in *Kuttervirus* phages.
• TSP subtypes can predict host ranges of uncharacterized *Ackermannviridae* phages.

## Graphical abstract

**Figure:**
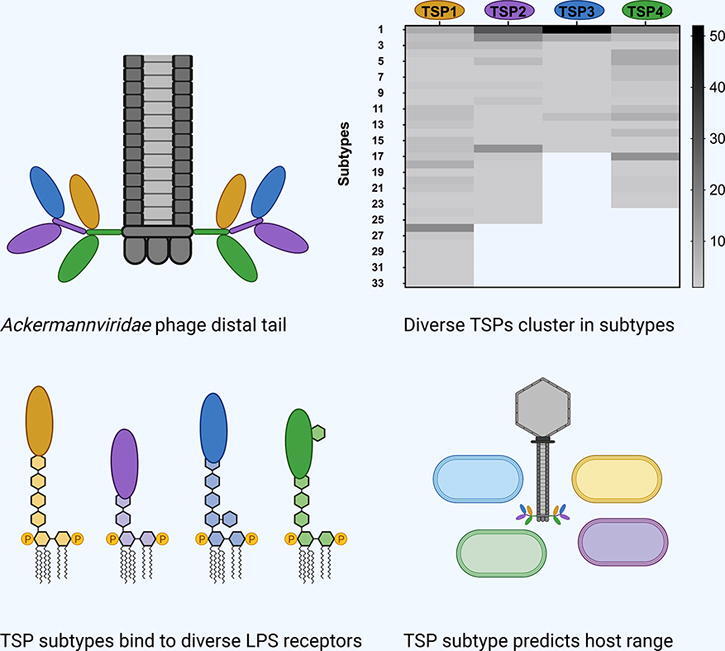

## 1 Introduction

Bacteriophages (phages) are viruses that specifically infect bacteria. As the first step in the infection cycle, tailed phages adsorb to the bacterial surface using their receptor-binding proteins (RBPs) located at the tip of the tail. Such RBPs often form long or short tail fibers or tail spike proteins (TSPs), allowing the phage to bind to specific receptors on the surface of the host bacterium. In contrast to tail fibers, TSPs often have enzymatic activity that bind and degrade polysaccharides structures such as lipopolysaccharides (LPS) or capsular polysaccharides (CPS) [1], [2], [3]. In the case of LPS, some TSPs recognize the core oligosaccharides, but more frequently they bind to the external moiety of LPS; the O-antigen [4]. While the core oligosaccharides of LPS are conserved, the O-antigen consisting of a repetitive oligosaccharide of three to five sugars are highly diverse even within a bacterial species. For example, more than 185 different O-antigens have been identified in *E. coli* creating high surface diversity within this species [5]. As the binding of the TSP to the O-antigen is highly specific, phages encode highly diverse TSPs to match the bacterial O-antigen diversity [6].

**Fig. 1:**
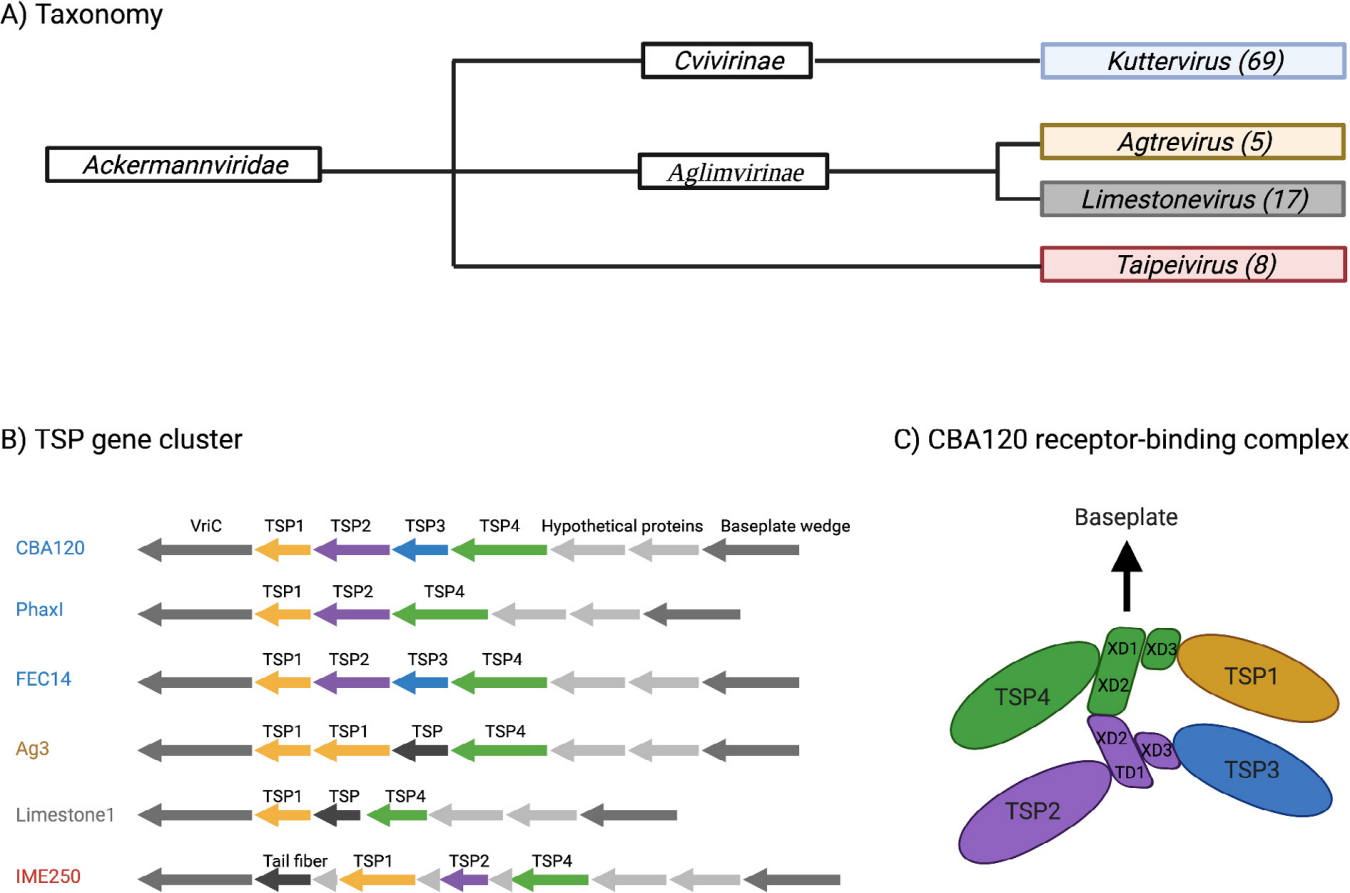
**Taxonomy of the *Ackermannviridae* family and the tail spike protein (TSP) gene clusters.** A) Taxonomy of the *Ackermannviridae* family with the subfamilies and genera. The number of phages analyzed in this study within the respective genera are presented in the parentheses. B) The organization of the TSP gene clusters in different *Ackermannviridae* phages. Blue: *Kuttervirus* phages, olive-yellow: *Agtrevirus* phage, grey: *Limestonevirus* phage, red: *Taipeivirus* phages. TSP genes were colored according to annotation in GeneBank. TSP1: Yellow, TSP2: Purple, TSP3: Blue, TSP4: Green, Not specified: Black. B) Receptor-binding complex consisting of the four TSPs in kuttervirus CBA120 proposed by Plattner et al. Abbreviation: TSP, tail spike protein and VriC, Virulence-associated protein.

While most phages only express one RBP, some phages express several RBPs that recognize different receptors thereby allowing the phage to infect multiple hosts even if the surface is highly variable [7], [8], [9]. Phages previously belonging to the *Viunaviruses* but now part of the *Ackermannviridae* family are known to encode multiple TSPs and infect a wide range of Gram-negative bacteria [10]. At the time of writing, the *Ackermannviridae* family consists of two subfamilies; *Cvivirinae* and *Aglimvirinae,* as well as the *Taipeivirus* genus [11] (Fig. 1A). The *Kuttervirus* is the only genus of the *Cvivirinae* subfamily and contains phages classified as either *Salmonella* or *E. coli* phages [12], [13]. The *Aglimvirinae* subfamily can be divided into the *Agtrevirus* and *Limestonevirus* genera that comprise phages infecting *Shigella, Enterobacter* and *Salmonella,* or *Dickeya*, respectively [14], [15]. Finally, the *Taipeivirus* genus include phages that infects *Klebsiella, E. coli* and *Serratia* [1], [16], [17]. Most phages belonging to the *Ackermannviridae* family express four TSPs (TSP1 to 4), however, some phages of the *Kuttervirus* and *Limestonevirus* genera only contain three TSP genes (Fig. 1B) [1], [14], [15], [18], [19], [20].

Even though the protein sequences of TSPs are highly diverse, their structure is conserved, forming a stable homotrimer in which each monomer displays right-handed β-helices. While the N-termini of TSPs, called the head-binding domain allows binding to the baseplate, the C-terminus contains a catalytic module domain that facilitate binding and degradation of the receptor as well as an intramolecular chaperone domain aiding in trimerization of the proteins [2], [20], [21], [22], [23], [24]. A recent study of the four TSPs encoded by *E. coli* phage CBA120 belonging to the *Kuttervirus* genus showed that the N-termini of the four TSPs were distinct and facilitate interactions between the TSPs to form a receptor-binding complex [20]. In CBA120, the N-termini of all four TSPs contains similar tandem domains called TD1 and TD2 with the exception of TSP2 that only contain TD1. In addition, TSP2 and TSP4 contains two and three XD domains (XD1-3), respectively, upstream of the tandem repeats that are structurally similar to the first three domains found in Gp10 in *E. coli* phage T4. In phage T4, the *gp10* encodes a baseplate protein containing four domains that forms a complex with other baseplate proteins (Gp11 and Gp12), which are responsible for attachment of the short tail fibers (Gp12) to the baseplate [25]. In CBA120, Plattner et al. proposed that TSP4 interact with the baseplate through the first 80 amino acid residues and the XD1 domain. The XD2 of both TSP2 and TSP4 are believed to interact with each other, allowing interaction between the two proteins. TD1 of TSP1 and TSP3 interact with the XD3 of TSP4 and TSP2, respectively. All these interactions are believed to be necessary for forming the receptor-binding complex [20] (Fig. 1C).

In CBA120, each of the four TSPs are able to recognize and bind different O-antigen moieties on the surface of *Salmonella* and *E. coli* [3], [20]. Individual purified TSPs showed that while TSP1 binds and degrades the O:21O-antigen on *Salmonella*, TSP2, TSP3 and TSP4 recognize O:157, O:77 and O:78 on *E. coli* strains, respectively [3], [20]. While it is assumed that *Ackermannviridae* phages are able to recognize different receptors, like CBA120, most of these phages have only been assigned to one host, mainly because of lack of extended host range analysis. In addition, due to the presence of the multiple TSPs, it is not well understood which TSP is responsible for the recognition of the assigned hosts, even when an extended host range analysis has been performed [26], [27], [28]. While some studies of *Ackermannviridae* phages indicate that these phages encode very diverse sets of TSPs [29], [30], other studies have demonstrated that some TSPs could be conserved within the different genera. An example is the TSP encoded by *orf169* of kuttervirus FEC14 which shows 99.5% identity to *orf211* encoding TSP2 in kuttervirus phage CBA120 [18]. Also, a recent study of limestonevirus phage PP35 showed that *orf156* was identical to several TSPs expressed by other *Limestonevirus* phages [31]. These studies suggest that TSPs in the *Ackermannviridae* family might be conserved, which could explain why different phages are able to infect the same hosts. Here we aim to reveal the diversity of TSPs encoded by *Ackermannviridae* phages by performing a comprehensive *in silico* analysis of these proteins in 99 phages. We found that different TSPs can be assigned into subtypes that correlate with the phage genus within the *Ackermannviridae* family. Furthermore, the TSP subtypes allowed us to predict host recognition of diverse TSPs. Finally, we experimentally determined the host recognition of three TSPs encoded by the *Salmonella* phage S117 (hereafter S117 or kuttervirus S117) belonging to the *Kuttervirus* genus and could confirm that phages expressing TSPs of the same subtypes recognize the same hosts. Our work demonstrates that by investigating TSP similarity and determining the host recognition of a few TSPs, it is possible to assign hosts for numerous uncharacterized phages.

## 2 Materials and methods

### 2.1 Bioinformatics analysis

To investigate the diversity of the TSPs in the *Ackermannviridae* family, all available genomes at the GenBank database were extracted (retrieved 24.11.2020). For the analysis, we searched for the well-conserved *vriC* gene that is present upstream of the TSP gene cluster. We identified this in 99 phages and afterwards, we numbered the TSPs 1 to 4 according to their N-terminal sequence similarity of the four TSPs in *Kuttervirus* CBA120. All multiple amino acid alignments were done in CLC Workbench 21.0.3 (Qiagen Digital Insights, Aarhus, Denmark) with the default settings; Gap cost 10, gap extension cost 1, end gap cost: as any others and alignment mode: very accurate. Pairwise comparison of the alignment allowed for determination of the percent identity of the TSPs. TSPs with 75% or more identity were assigned into TSP subtypes. Each predicted TSP was verified using HHPRED [32]. Furthermore, to identify the structural domains in the N-termini we used one representative TSP of each subtype for the HHPRED analysis because of the high sequence similarity of the TSPs in each TSP subtype.

Whole genome alignment of the 99 *Ackermannviridae* phage genomes was done in CLC Workbench 21.0.3 (Qiagen Digital Insights, Aarhus, Denmark) with the default settings (minimum initial seed length: 15, allow mismatches in seeds: yes and minimum alignment block length: 100). Pairwise comparison of the analysis was conducted to create a heatmap displaying the average nucleotide identity (ANI) using default settings (table types: ANI, distance measure: euclidean distance and linkage criteria: complete linkage).

### 2.2 Appendices

Appendix A: overview of all phages analyzed with accession numbers, gene ID of the identified TSPs and the categorized TSP subtypes for each TSP. Appendix B-E: Overview of the TSP1 to TSP4 pair wise comparison and the number of phages expressing the specific TSP subtypes. Appendix F: overview of all bacterial strains used.

Mendeley data: https://data.mendeley.com/datasets/k6pynfdzch/2

### 2.3 Bacteria and phage

*Salmonella* strains for phage propagation, bacterial strains for host range analysis, strains used for TSP cloning and purification and *Salmonella* mutants for TSP3 receptor determination are listed in the Appendix F. *Salmonella* phage S117 (GenBank accession number MH370370.1) [33] and its four TSPs were used in the experimental work.

### 2.4 Phage propagation

Phage S117 was propagated on *S. Typhimurium* LT2c strain, which is cured from prophages *gifsy-1*, *gifsy-2*, *fels-1* and *fels-2*. First, a single colony from LT2c was inoculated into LB media (Lysogeny Broth, Merck, Darmstadt, Germany) and was grown to the exponential phase at 37 °C and 180 rpm. Then, a previous phage stock of S117 (4*10^10^ pfu/ml) was 10-fold diluted and the dilutions 10^−3^ to 10^−5^ were individually mixed with 100 µL of the LT2c exponential cell culture. The cell-phage suspension was added to 3.3 mL molten top agar (LBov; LB broth with 0.6% Agar bacteriological no.1, Oxoid) and poured onto a LA plate (LB with 1.2% agar). After the plate was dried for 45 min in a laminar hood, the plate was incubated aerobically at 37 °C overnight. The next day, 5 mL of SM buffer (0.1 M NaCl, 8 mM MgSO_4_·7H_2_O, 50 mM Tris-HCl, pH 7.5) was added to the plate with abundant plaques and the plate was incubated for 24 h at 4 °C and 50 rpm. The phage suspension in SM buffer was collected and centrifuged for 15 min at 11000 rpm. The supernatant was filtered twice with a 0.2 µM filter and a 10-fold serial dilution of the new phage stock was prepared to determine the phage titer by phage plaque assay.

### 2.5 Phage plaque assay

To determine the phage titer, host range and to verify the receptor identified for for TSP3, 10-fold serial dilutions (10^−1^ to 10^−8^) of the newly prepare S117 phage stock in SM buffer were prepared. Three times 10 µL of each phage dilutions were spotted onto bacterial lawns and plates were dried for 45 min in a laminar hood. The plates were incubated overnight at 37 °C and the following day, plaques were counted and the plaque forming unit per ml (pfu per ml) was calculated. The efficiency of plating was calculated by comparing the pfu/mL of the S117 screened strains (Appendix F) with the propagated strain *Salmonella* Typhimurium LT2c.

### 2.6 DNA extraction and purification of phage DNA

The methods for DNA extraction and purification have been described elsewhere [33]. Briefly, the S117 phage stock was filtered three times with 0.2 µM filters. RNAse (10 µg/mL) (Thermo Fischer Scientific, Waltham, MA, USA) and DNAse (20 µg/mL) (Thermo Fischer Scientific) was added to the lysate and incubated for 20 min at 37 °C in a thermoshaker (500 rpm, Eppendorf, Germany). After incubation, EDTA (pH8) (20 mM) (Thermo Fischer Scientific) and Proteinase K (50 µg/mL) (Thermo Fischer Scientific) were added followed by incubation for 2 h at 56 °C. A sample was run on an 1% agarose gel to confirm the release of the DNA from the phage capsids. Phenol (Fluka), phenol–chloroform-isoamylalcohol (25:24:1, Ambion) and three rounds of chloroform-Isoamylalcohol (24:1) were used for DNA purification. The DNA was precipitated with 0.1 vol 3 M sodium acetate (pH 5.5) (Thermo Fischer Scientific), Glycogen (0.05 µg/µL) (Thermo Fischer Scientific) and 2.5x ice-cold 99% ethanol and incubated at −18 °C for 72 h. The precipitated DNA was centrifuged at 4 °C for 20 min at 12000 rpm and the pellet was washed three times with ice-cold 70% ethanol. Afterwards, the pellet was resuspended in 10 mM Tris-HCl (pH 8). The quality of the purified DNA was confirmed with Nanodrop (Thermo Fischer Scientific) and gel electrophoresis. The concentration was estimated with Qubit (Thermo Fischer Scientific).

### 2.7 TSP cloning and purification

The four TSPs of phage S117 were individually cloned into pET-28a (+) with a purification His-tag downstream of the TSP genes by using In Vivo Assembly (IVA) cloning described previously [34]. Briefly, each *tsp* gene (TSP1 (2313 bp), TSP2 (2766 bp), TSP3 (2097 bp) and TSP4 (3504 bp)) were individually amplified with primers carrying overhang homologous to the pET-28a (+) in a PCR tube also containing the pET-28a (+) vector and primers for amplifying the vector. The homologous primer overhangs allow for homologous recombination upon transformation. The TSP genes were cloned into the multiple cloning site between the HinCII and Eco52KI restriction sites. Phusion® High-Fidelity DNA polymerase (NEB©) was used to amplify the TSP genes and the pET-28a(+). PCR primers (Supplementary Table 1) were designed with SnapGene® and ordered from TAG Copenhagen A/S. The PCR reaction followed the manufactures description with 1 ng S117 DNA and 1 ng pET-28a(+) as templates with 0.1 µM primers. Subsequently, 1 μL FastDigest DpnI enzyme (Thermo Fisher Scientific) was added to the PCR sample and incubated for 15 min incubation at 37 °C to digest the methylated DNA template. The DpnI-digested PCR sample was propagated into Stellar™ Competent Cells (Takara Bio) using the manufactures protocol except for extending the heat shock to 60 sec. The TSP-inserted-plasmid (pET_TSP1 to 4) was isolated with GeneJET Plasmid Miniprep kit (Thermo Scientific) from an overnight culture. PCR on the isolated plasmid was used to validate the presence of the insert and successful plasmid constructions were confirmed by Sanger sequencing (Eurofins Genomics).

pET_TSP1 to 4 were transformed into electrocompetent *E. coli* BL21 cells (Agilent Technologies). To express the TSPs, a single colony of the strain containing each plasmid was inoculated into LB medium at 37 °C and 170 rpm. At optical density (OD_600_) of 0.6 the culture was cooled on ice prior to addition of 0.5 mM isopropyl-β-D-thiogalactopyranoside (IPTG) to induce the TSP expression. The culture was then incubated at 16 °C and 110 rpm for 16–18 h. The cells were harvest by centrifugation for 10 min at 12000 rpm and stored in the freezer until further processed. The pellet was resuspended in lysis buffer (0.5 M NaCl, 20 mM Na_2_HPO_4_, 50 mM Imidazole, pH 7.4) and the cells disrupted on ice by sonication (Bandelin Sonopul HD 2070 homogeniser). The lysate was filtered twice with 0.22 µM filters before the His-tagged TSP was purified using HisGraviTrap™ (GE Healthcare) according to the manufacture’s description with elution buffer (0.5 MNaCl, 20 mM Na_2_HPO_4_, 0.5 M Imidazole, pH 7.4). Amicon® Ultra-15 Centrifugal filter units with 50 kDa cut-off (Merck Milipore) was used to exchange the protein solutions to 20 mM HEPES (pH 7.4). Protein concentration was measured with Qubit™ Protein Assay Kit (Q33211) with a Qubit 2.0 Fluorometer (Invitrogen, Q32866).

### 2.8 Spot assay

To determine the host recognition for each of the four TSPs a spot assay was carried out. 100 µL of a bacterial overnight culture was added to 4 mL top agar and poured onto a LB agar plate. Afterwards 1.5 µg of the four TSPs was spotted onto the lawn and the protein buffer (20 mM HEPES) was used as a negative control. The plates were incubated overnight at 37 °C and the next day the plates were checked for the presence of a translucent clearing zone in the spots with purified TSP proteins indicating TSP degradation of the receptor.

### 2.9 TSP inhibition assay

To validate the spot assay, we determined if the TSPs where able to inhibit the infection of S117 on the respective hosts. *S. Minnesota* (JEO2), *E. coli* O157:H7 (NTCT12900) and *S. Typhimurium* (LT2c) was used as strains for TSP1, TSP2 and TSP3 respectively. The bacteria were grown to OD_600_ of 0.3 and put on ice where the four TSPs (0.5 mg/mL) or nothing were added to 100 µL of the bacteria. The TSP-bacteria suspension was preincubated at 37 °C for 20 min before being added to top-agar and poured onto a LB agar plate. After the bacterial lawn had dried, three times 10 µL of a serial dilution (10^−1^ to 10^−8^) of S117 was spotted onto the lawn. The plates were incubated overnight at 37 °C and the next day plaques were counted and the pfu/ml calculated. The efficiency of plating was calculated by comparing the pfu/mL of the S117 sensitive *Salmonella* Typhimurium LT2c mutant strains with the wild type.

### 2.10 Statistical analysis

All results were conducted in three independent experiments where the mean standard deviations are shown in the graphs. One-way ANOVA was used to access the statistical significance.

## 3 Results

### 3.1 Tsps subtypes are associated with phages genus

All classified *Ackermannviridae* phages encode multiple TSPs located in a gene cluster flanked by a conserved virulence associated protein gene (*vriC*) and a baseplate wedge gene (Fig. 1B). To investigate the diversity of the TSPs in the *Ackermannviridae* family, we extracted all available genomes (1 3 3) classified as such from NCBI and searched for the gene cluster using the conserved *vriC* gene as a reference. This approach allowed us to identify the *vriC* gene and the complete downstream TSP cluster in 99 genomes, whereas 34 genomes were discarded from further analysis. Of these, four phages contained the *vriC* gene, but the downstream genes could not be classified as full length TSPs, whereas 30 genomes were unclassified *Ackermannviridae* phages not encoding the *vriC-tsp* loci. We then performed homolog detection and structural prediction by HHPRED [32] of all individual TSP proteins in the 99 genomes to validate that the genes in fact encoded TSPs (results not shown). Among the 99 phages choosen for futher analysis, 69 belonged to the *Kuttervirus* genus, five to the *Agtevirus* genus, 17 to the *Limestonevirus* genus and eight to the *Taipeivirus* genus. A detailed overview of all phages analysed as well as the protein ID of the defined TSPs when applicable are shown in Appendix A.

To investigate the conservation and similarity of the *Ackermannviridae* TPS, we first assigned all TSPs as either TSP1, TSP2, TSP3 or TSP4 based on amino acid similarity to the N-termini of the four well-characterised TSPs of *Kuttervirus* phage CBA120. We identified: 97 TSP1s, 99 TSP2s, 78 TSP3s and 98 TSP4s (Appendix A). We then performed multiple protein alignement of all TSP1, TSP2, TSP3 and TSP4. This analysis showed that the similarity of the TSPs ranged from 5 to 100% identity, demonstrating that the TSPs in the *Ackermannviridae* family can be highly diverse (Appendix B-E). Interestingly, some phages encoded highly similar TSPs with 75–100% identity, yet these TSPs showed only limited similarity (7–30%) to other TSPs assigned as the same type. For instance, four phages encoded TSP1s that were 99–100% identical, whereas the remaining TSP1s only showed ≤ 29% identity to these four TSPs (Appendix B). It was previously shown that TSPs sharing sequence similarity could be used to predict the receptor for the TSP [20], [35]. For instance, TSP4 of kuttervirus CBA120 were 74–79% identical to TSPs from three G7C-like phages. The authors showed that TSP4 of CBA120 recognize the same receptor as the three G7C-like phages [20]. Thus, we used this information to further assign the TSPs into subtypes using 75% identity as a cut-off value, however, the majority of the TSPs in the specific subtypes were geneally more than 95% identical (Appendix B-E). Accordingly we could identify 33, 25, 16 and 23 distinctive subtypes of TSP1, TSP2, TSP3 and TSP4, respectively.

**Fig. 2:**
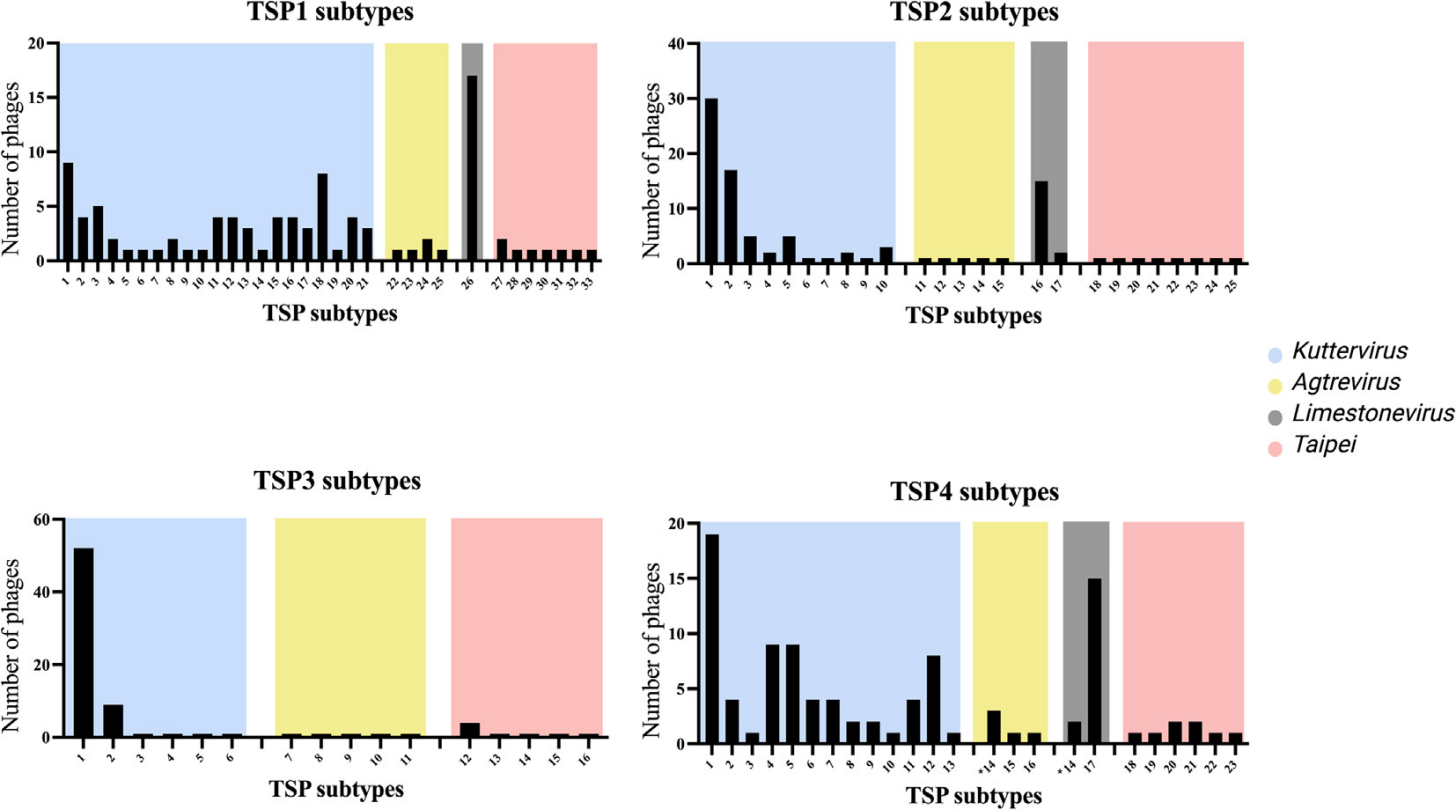
**TSP subtypes in the *Ackermannviridae* family correlate with the phage genus.** TSP genes were identified in the phage genomes and grouped into TSP1, TSP2, TSP3 and TSP4 and aligned. TSPs that were 75% or more identical were grouped into subtypes. The number of phages grouped into the individual TSP subtypes are illustrated on the y-axis. Blue: TSP subtypes expressed by *Kuttervirus* phages, Olive-yellow: TSP subtypes expressed by *Agtrevirus* phages, Grey: TSP subtypes expressed by *Limestonevirus* phages and Red: TSP subtypes expressed by *Taipeivirus* phages. Asterisk: TSPs in the TSP4-14 subtype are expressed by *Agtrevirus* and *Limestonevirus* phages. The figure was generated using Prism 9.

During our analysis, we observed that each TSP subtype could be associated with a specific phage genus with the only the exception of subtype TSP4-14 that was encoded by phages belonging to both the *Limestonevirus* and *Agtrevirus* genera (Fig. 2). We found that *Kuttervirus* phages express a more diverse set of TSP1, TSP2 and TSP4 subtypes compared to the other phages. For instance, 21 of 32 TSP1 subtypes identified were expressed by *Kuttervirus* phages (Fig. 2). On the contrary, only six different TSP3 subtypes were identified for *Kuttervirus* phages as 52 of the 69 *Kuttervirus* phages express subtype TSP3-1. In contrast to the *Kuttervirus*, all 17 *Limestonevirus* phages analysed express TSP1 belonging to the same TSP subtype (TSP1-26). Furthermore, 15 out of 17 *Limestonevirus* phages express the subtypes TSP2-16 and TSP4-17, demonstrating that the TSPs within this group of phages are highly conserved. Intrestingly, we did not identify a TSP3 in any of the *Limestonevirus* phage genomes. In the *Agtrevirus* genus, the five phages overall expressed more unique TSPs, as all of the five TSP2 and TSP3 grouped into separate subtypes. However, TSP1 of two phages grouped into TSP1-24, and four phages encoded TSP4-14, the same subtype as the *Limestonevirus* phages; RC-2014 and phiDP10.3 (Fig. 2). Similar to the *Agtrevirus* phages, the eight phages in the *Taipeivirus* genus also encode unique TSPs, as only one or a few phages encode TSPs of the same subtypes. Similar to the *Agtrevirus* phages, the eight phages in the *Taipeivirus* genus also encode unique TSPs, as only one or a few phages encode TSPs of the same subtypes.

The 69 of the 99 *Ackermanviridae* genomes analysed were *Kuttervirus* phages while only five, 17 and eight genomes belonged to *Agtrevirus* phages, *Limestonevirus* phages and *Taipeivirus* phages, respectively. Thus, we speculated that the larger diversity observed in the TSPs for the *Kuttervirus* and the highly similar TSPs found in *Limestonevirus* may results from the overall genomic diversity found within the representatives analysed for each genus. We therefore performed whole genome analysis of all 99 phages genomes and found that the *Limestonevirus* phages had a average nucleotide identity (ANI) between 96 and 100%, whereas the phages in the other genera were not as similar (*Kuttervirus* phages: ANI of 88–100%, *Agtrevirus* phages: ANI of 89–96% and *Taipeivirus* phages: ANI of 86–97%) (Fig. S1). However, phages in the *Kuttervirus* genus that shared an ANI of 99–100% also expressed TSPs categorized into the same subtypes. Thus, as the genomes of the *Limestonevirus* phages analysed are highly conserved this may explain why these phages express similar TSPs compared to the other phage genera. Overall, we found that phages in the *Ackermannviridae* family express highly diverse TSPs that can be grouped into subtypes based on similarity. Furthermore, each TSP subtype were specifically associated with the phage genera.

### 3.2 The N-terminal XD domains of TSP4 are conserved within *Ackermannviridae* phages

It was previously suggested that the XD domains located in the N-termini of TSP2 and TSP4 are essential for forming the branched receptor-binding protein complex as observed in phage CBA120 [20]. To investigate if such receptor-binding complex are preserved in *Ackermannviridae* phages, we analyzed the N-termini of all TSP subtypes for homology detection and structure prediction using HHPRED. In phage CBA120 TSP2 and TSP4, the XD domains are located within the first 300 and 500 residues, respectively, thus we used these regions for our analysis [20].

For all TSP2 subtypes, we found high similarity to the N-terminus of TSP2 of CBA120 (PDB ID 6W4Q) with a probability ranging from 98.16 to 100% and e-values spanning from 4e^−11^ to 7.7e^−151^ (Table 1). For some TSP2 subtypes, only the first 85 residues were similar to TSP2 of CBA120 (Table 1), which correlates with the proposed XD2 domain that spans the first 88 residues in TSP2 of CBA120. The XD domains of TSP2 of phage CBA120 were identified based on structural similarity to domain two and three in Gp10 of phage T4 (PDB ID 5XH2). We did, however, not detect such similarity in any of the TSP2 subtypes. Yet, all TPS2 subtypes were structurally similar to TSP2 of CBA120, suggesting an overall similar fold of the N-termini of TSP2 subtypes in *Ackermannviridae* phages. Analysis of the TSP4 subtypes showed structural similarity to the Gp10 protein (PDB ID 5XH2) encoded by phage T4 as previously observed for TSP4 of CBA120 [20] (Table 2). In addition, we also found similarity to Gp9 of T4 (PDB ID 12SE) from the first residue to residue 217 with a general higher probability than T4 Gp10 (Table 2). Yet, it was previously shown that Gp10 and Gp9 have similar folding in the N-termini, which explains our findings [25]. While all TSP4s have structural similarity to the first two domains in T4 Gp10, hence XD1 and XD2 domains, there were no structural similarity to domain 3 (XD3) of Gp10 in some TSP4s (Table 2). We did not find any distinctive structural domains in the N-termini of TSP1 and TSP3. Overall, our analysis showed that the structural domains XD1 and XD2 proposed to be necessary for hinging the receptor-binding complex together are preserved in all *Ackermannviridae* phages, whereas the XD3 domain interacting with TSP1 and TSP3 are not always preserved.

**Table 1:** HHPRED analysis overview of the N-terminal structural similarity in the TSP2 subtypes.

^a^ Subtypes expressed by phages belonging to *Kuttervirus* (blue), *Agtrevirus* (yellow), *Limestonevirus* (grey), *Taipeivirus* (red).

**Table 2:** HHPRED analysis overview of the N-terminal structural similarity in the TSP4 subtypes.

^a^ Subtypes expressed by phages belonging to *Kuttervirus* (Blue), *Agtrevirus* (yellow), *Limestonevirus* (grey), *Taipeivirus* (red).

### 3.3 Conserved receptor binding domains are found in TSP1, TSP3 and TSP4 subtypes suggesting recombination events

**Fig. 3:**
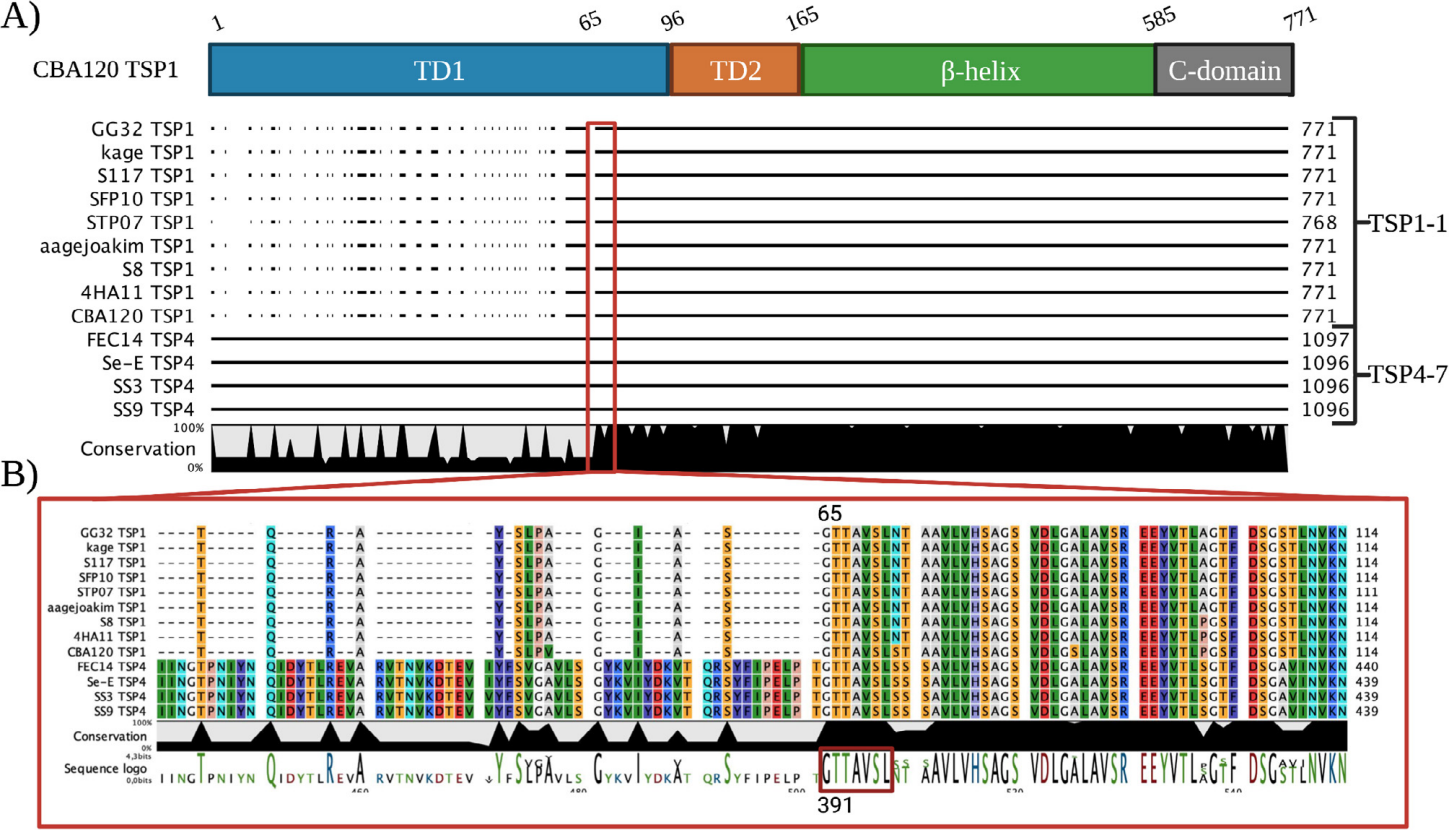
**The receptor binding domains can be swapped between TSPs in the *Kuttervirus* phages.** A) All TSP1s belonging to subtype 1–1 were aligned with all TSP4-7. B) Zoom in on the start positions of similarity of the TSPs. The alignment shows a conserved sequence motif that starts at the same amino acid position 65 and 391 for TSP1s and TSP4s, respectively. The structural domains of CBA120 TSP1 was used as a reference.

While the N-terminal regions of each of TSP1, TSP2, TSP3 and TSP4 were highly conserved, the C-terminal regions were highly diverse, even among TSP subtypes (Fig. 2 and Appendix B-E). However, when we aligned all TSP1, TSP2, TSP3 and TSP4, we observed that among *Kuttervirus* phages, some TSP1 and TSP3 amino acid sequences were similar to TSP4 sequences except for the XD domains found in TSP4 subtypes (Figs. 3 and S1). More precisely, sequence similarity was observed between TSP1-1 and TSP4-7 subtypes (Fig. 3A), TSP3-3 and TSP4-2 subtypes (Fig. S2A), and TSP3-1 and TSP4-8 subtypes (Fig. S2B). The similarity starts at amino acid position 65 for the TSP1s and TSP3s and position 391 for the TSP4s (Fig. 3B). When we aligned the sequences from position 65 and 391, respectively, TSPs belonging to subtype TSP1-1 and TSP4-7 were 92–100% identical, TSPs belonging to TSP3-3 and TSP4-2 were 93–100% identical, and TSPs belonging to TSP3-1 and TSP4-8 subtypes were 91–100% identical (results not shown). The results suggest that the same receptor binding module can be located on different TSP types.

It has previously been suggested that the β-helix, hence the receptor binding module, can be swapped among TSPs thereby changing the host range of the phage [2], [36]. When using the structural domains defined in the CBA120 TSP1 as a reference, we identified a conserved sequence motif; GTTAVSL at the distal part of the TD1 domains of subtypes in the TSP1-1, TSP4-7, TSP3-3, TSP4-2, TSP3-1, TSP4-8 subtypes showing similarity across TSPs (Fig. 3B). We therefore speculated if the end of the TD1 domain could form a conserved site, allowing recombination between the TSPs. To further investigate if this region is conserved in all TSP1, TSP2, TSP3 and TSP4s, we extracted the sequences spanning the TD1 domain from all TSP subtypes (region 1–96 aa in TSP1 and TSP3, region 157–257 aa in TSP2 and region 344–421 aa in TSP4 [20]) and aligned them. Firstly, across phage genera only little sequence similarity of the TD1 domains of TSPs was observed (results not shown). Secondly, the sequence spanning the TD1 domain in *Kuttervirus* TSP2 subtypes were not similar to the TD1 domain in any other *Kuttervirus* TSP subtypes (results not shown). However, when we aligned sequences spanning the TD1 domains of all *Kuttervirus* TSP1, TSP3 and TSP4 subtypes, we found an overall conservation, where the GTTAVSL motif was present in many of the TSP subtypes with the exception of TSP4-13 where only limited similarity was found (Fig. S3). These observations further suggest that a conserved site in the TD1 domains of TSP1, TSP3 and TSP4 in the *Kuttervirus* phages may be involved in swapping the receptor binding regions of these TSPs, thus providing a mechanism for alternate host recognition. Overall, our results demonstrate sequence similarity in the β-helix and C-terminal domains between some TSP1, TSP3 and TSP4 subtypes in the *Kuttervirus* genus. Furthermore, we identified a conserved site in the TD1 domains of these TSPs, which potentially could promote recombination events among TSPs in this genus.

### 3.4 TSP subtypes can be used to predict hosts bacteria of *Ackermannviridae* phages

Our *in silico* analysis showed that the majority of the *Ackermannviridae* phages encode four TSPs, which most likely recognize different bacterial receptors, suggesting that phages in this family have a wide host range. In general, phages beloning to the four *Ackermannviridae* genera have been shown to infect diverse Gram-negative bacteria [1], [14], [33], [37]. However, for most of the phages, a detailed host range analysis has not been conducted. In addition, the respective hosts for each of the TSPs have only been shown for kuttervirus CBA120 [20]. To investigate if we could associate TSP subtypes with host recognition, we used published data of TSPs already characterized in terms of receptor recognition.

**Fig. 4:**
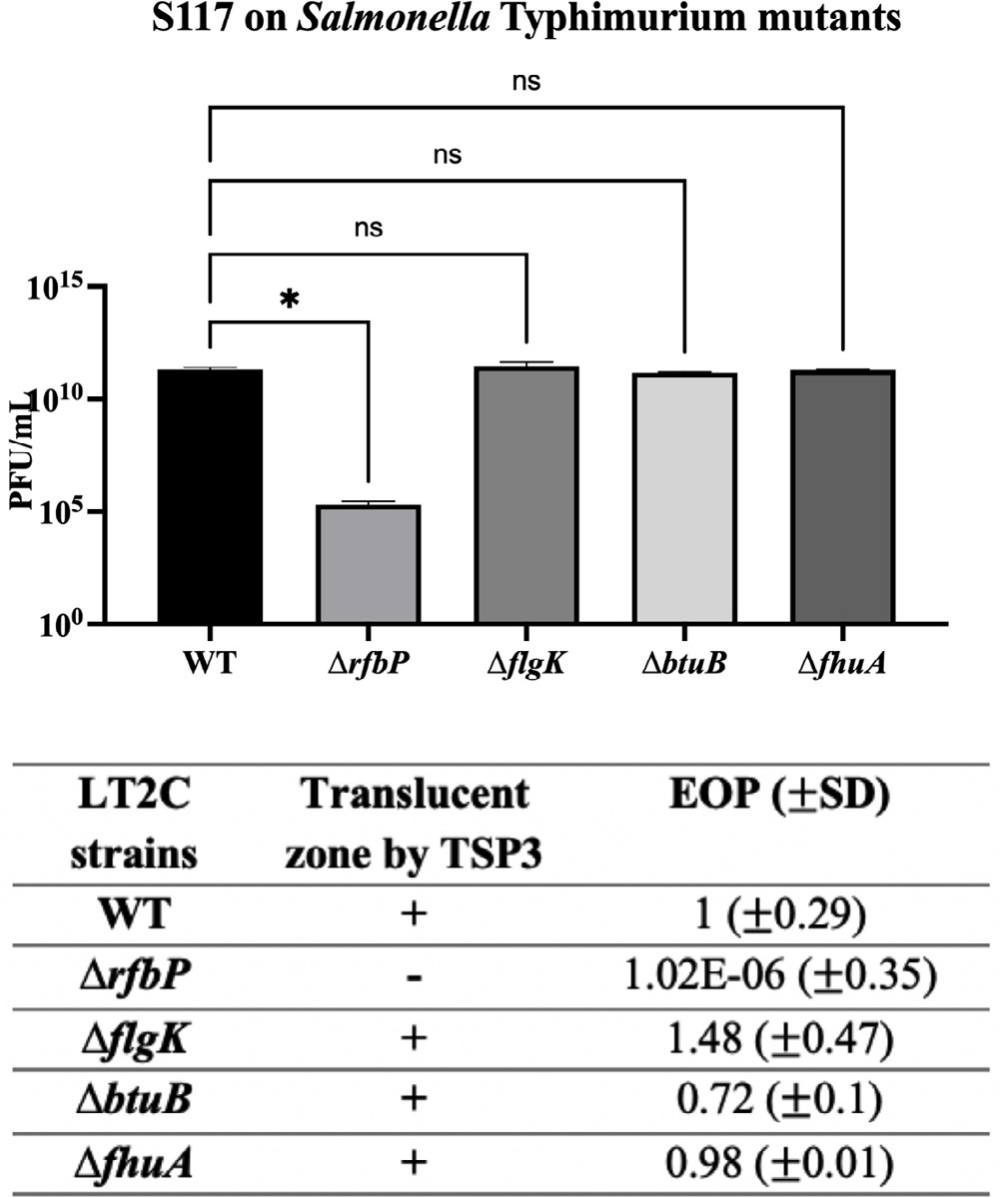
**TSP3-1 was not able to produce a translucent zone on an LPS mutant (**Δ***rfbP*).** Phage S117 and TSP3-1 were spotted on *Salmonella.* Typhimurium (LT2c) mutant strains lacking known phage receptors; O-Ag (*rfbP*), flagella (*flgK*), ferrichrome transporter (*fhuA*) and vitamin B_12_ transporter (*btuB*). The experiment was carried out in triplicates and the error bars represent the standard deviation. The graph was generated in prism9 where the p-values were calculated using the ordinary one-way ANOVA. +: appearance of a translucent zone, -: no zone.

TSP1 of kuttervirus CBA120 was assigned to the TSP1-1 subtype and a total of 9 phages encode this subtype (Fig. 2). TSP1 of CBA120 recognize the O:21O-antigen of *Salmonella* Minnesota, thus we predict that all phages expressing the TSP1-1 subtype also recognize the O:21 antigen (Table 3). In addition, our bioinformatic analysis showed that TSP1-1 was almost identical to TSP4-7 except for the N-terminal region containing the XD domains promoting hinging of the receptor binding complex together (Fig. 3). It is therefore likely that the four phages encoding TSP4-7 subtypes recognize *Salmonella enterica* O:21 serotypes as well (Table 3). TSP2 of CBA120, belonging to the TSP2-1 subtype, binds and degrades the O:157O-antigen on Shiga toxin (Stx)-producing *Escherichia coli* (STEC) strains, suggesting that all 32 phages expressing TSP2-1 s recognize the O:157O-antigen [20]. This is also supported by the fact that 11 out of the 32 phages expressing TSP2s belonging to the TSP2-1 subtype are already known to infect O:157 *E. coli* strains [12], [18], [19], [30], [33], [38], [39], [40], [41]. The last two TSPs of CBA120, TSP3 (TSP3-4) and TSP4 (TSP4-2), recognize *E. coli* O:77 and O:78, respectively. While CBA120 is the only phage that express a TSP3-4 subtype, CBA120 and 3 other *Kuttervirus* phages express TSP4-2 and thus may recognize *E. coli* O:78. In addition, we showed that the C-terminal domains of TSP4-2 and TSP3-3 subtypes are almost identical, suggesting that TSPs belonging to the TSP3-3 subtype also recognize *E. coli* O:78 (Fig. 4 and Table 1).

**Table 3:** Host range predictions of Ackermannviridae phages based on the TSP subtypes.

**Identified/predicted host**	**TSP subtype(s)**	**Phages in total**	***Ackermannviridae* phages expressing TSPS in the same TSP subtype**
*S*. Ruiru and *S*. Minnesota O:21	TSP1-1	9	GG32, Kage, S117, SFP10, STP07, Aagejoakim, S8, 4HA11 and CBA120 [20]
	TSP4-7	4	FEC14, SS3, SS9 and Se-E
*E. coli* O:157	TSP2-1	32	Rabagast, Moki, Heyday, SeHz-1, S118, S115, Se-U, Pa-sanjiao, S8, Se-D, Se-B, BSP101, Kage, SFP10, STP07, Se_EM1, GG32, 4HA11, Se-E, FEC14, S117, EP75, SJ3, PhaxI , FSL_SP-063, FSL_SP-029, STML-13–1, ECML-4, Aagejoakim and CBA120 [20]
*S*. Typhimurium, *S*. Derby, S. 4.12:i:-, S. 4.5.12:i:-, *S*. Enteritidis, *S*. Goettingen O:4 and O:9	TSP3-1	52	TSP3-1:PS5, Marshall, Mooltan, STML-13–1, Matapan, Maynard, FSL-SP-029, FSL-SP-063, SJ2, Sal157lw, Bering, ISTP3, Se-H, BSP101, Se-I, Se-J, Se-N, Se-S, SP1, Dinky, SenASZ3, Sh19, Moki, S117, Pa-sanjiao, S115, S118, Se-U, SFP10, Aagejoakim, Kage, Rabagast, 4HA11, SeHz-1, Se-E, Heyday, S8, GG32, STP07, Se-F, Se-G, SenALZ1, SeTs-2, Se-B, Se-D, Pertopsoe, SS3, SeSz-1, Se_AO1, Se_EM3, Se_EM1, and Se_EM4
	TSP4-8	2	SE14 and mane
*E. coli* O:77	TSP3-4	1	CBA120 [20]
*E. coli* O:78	TSP4-2	4	BSP101, S8, PhaxI and CBA120 [20]
	TSP3-3	1	FEC14
*S.* Typhimurium O:4	TSP3-2	9	SJ3, EP75, LPSTP4, Mutine, PM10, Chennai, SenM-2 and barely and Det7 [21]
*S.* Anatum O:3	TSP2-10	3	SenM-2 and barely and Det7 [42]
*E. coli* O:18A	TSP1-3	5	SeTs-2, Se-F, Se-G and SenALZ1 and EP75 [46]
*Dickeya* Solani and *Lelliottia* F154	TSP1-26	17	PhiDP23.1, Coodle, XF4, PhiD3, Kamild, Limestone1, JA15, PhiDP10.3, RC-2014, Ds16CZ, Ds9CZ, Ds5CZ, Ds3CZ, Ds23CZ, Ds20CZ and Ds25CZ and PP35 [31]
*Klebsiella* KN2 capsular	TSP2-18	1	0507-KN2-1 [17]

The receptors of TSP2 (TSP2-10) and TSP3 (TSP3-2) of *Kuttervirus* Det7 have previously also been determined [42], [43]. TSP2 of Det7 binds to the O:3O-antigen from *S. Anatum* [42]. Thus, we predict that the two phages; SenM-2 and Barely that also express a TSP2-10 subtype also recognize *S. Anatum*, which indeed has been shown for phage SenM-2 [43]. TSP3of phage Det7 binds and degrades the O:4O-antigen of *S. Typhimurium* [21], [44]. A total of nine phages express the TSP3-2 subtype, thus we predict that these nine phages bind to *S. Typhimurium* O:4*.* Indeed, *S. Typhimurium* has already been identified as a host of four of these nine phages (EP75, Mutine, PM10 and SenM-2) [29], [43], [45], [46]. A recent study of the four TSPs of EP75 showed that TSP1 in the TSP1-3 subtype recognize the O:18A O-antigen of *E. coli* [46]. It is, therefore, likely that the four other phages expressing this subtype recognize the O:18A O-antigen (Table 3).

The TSP1-26 of limestonevirus phage PP35 recognize the O-antigen; (→2)-β-D-6-deoxy-D-altrose-)1 → ) of its hosts *Dickeya solani* and *Lelliottia* sp. F154 [31]. Our analysis revealed that all *Limestonevirus* phages express TSP1-26, thus the results suggest that all phages in the *Limestonevirus* genus can bind and degrade this LPS. This is in accordance with the known hosts of almost all *Limestonevirus* phages [14], [31], [47], [48], [49], [50], [51], [52], [53]. Finally, TSP2 (TSP2-18) of taipeivirus 0507KN21 recognize the KN2 capsular polysaccharide [17]. This phage is the only *Ackermannviridae* phage expressing a TSP2 of that subtype (Table 3). Overall, we use our *in silico* analysis and already published experimental data to associate the presence of specific TSPs with potential hosts of phages belonging to the *Ackermannviridae* family. Revealing the TSP homology between phages in the *Ackermannviridae* family, allowed us to assign the receptors for many phages by only knowing the receptor recognition for a few TSPs.

### 3.5 Three TSPs of *Kuttervirus* phage S117 recognize specific hosts expressing different O-antigens

To further validate our host range predictions, we expressed and purified the four TSPs of kuttervirus S117 phage from our collection [33]. From an extensive host range analysis, we previously showed that S117 infects *E. coli* O:157 as well as several *Salmonella enterica* serotypes [33]. Our bioinformatic analysis assigned the four TSPs of phage S117 into the following subtypes: TSP1-1 (9 phages), TSP2-1 (32 phages), TSP3-1 (52 phages) and TSP4-1 (18 phages) and as indicated in brackets these subtypes are conserved in many other *Kuttervirus* phages (Fig. 2). Furthermore, the *in silico* analysis predicts that TSP1 and TSP2 of phage S117 target O:21 of *Salmonella* and O:157 of *E. coli*, respectively. Purified TSPs at 95°for 10 min ran as monomers during SDS-PAGE analysis, whereas sizes corresponding to TSP trimerization could be detected on the gels without heating, suggesting that the TSPs were correctly folded and thereby enzymatical functional (Fig. S4). These properties are a common characteristic for TSPs [54]. TSPs are known for their enzymatic activity towards their receptors, and when spotted on a bacterial lawn, a translucent zone appears as the TSPs degrade the polysaccharide receptor [17], [55], [56]. Purified TSPs as well as phage S117 were spotted on 65 *Salmonella enterica* strains, 76 *E. coli* strains and 7 other gram-negative strains to identify the host of each of the TSPs (Table 4). This allowed us to identify the TSP responsible for binding to a specific host and identified the O-antigen recognized by TSP1, TSP2 and TSP3, while host recognition for TSP4 could not be found. Our analysis showed that TSP1 was able to bind to different *Salmonella enterica* serovars belonging to O:21 serotype (Table 4), whereas TSP2 was indeed able to recognize *E. coli* O:157 strains (Table 4), in agreement with our *in silico* predictions (Table 3).

**Table 4:** **Summary of the host range analysis of S117 and its four TSPs.** Successful phage infection or detection of a translucent zone are in indicated in bold. A detailed description of the strains used can be found in Appendix F.

			**Infected or translucent zone/Tested strains**				
**Genus**	**Species or serovar**	**O-antigen**	**S117**	**TSP1-1**	**TSP2-1**	**TSP3-1**	**TSP4-1**
***Salmonella***	**Derby**	**O:4**	**2/17**	0/17	0/17	**2/17**	0/17
***Salmonella***	**Typhimurium**	**O:4**	**6/8**	0/8	0/8	**6/8**	0/8
***Salmonella***	**4.12:i:-**	**O:4**	**2/4**	0/4	0/4	**2/4**	0/4
***Salmonella***	**4.5.12:i:-**	**O:4**	**5/6**	0/6	0/6	**5/6**	0/6
*Salmonella*	Bradenburg	O:4	0/1	0/1	0/1	0/1	0/1
*Salmonella*	Bradford	O:4	0/1	0/1	0/1	0/1	0/1
***Salmonella***	**Dublin**	**O:9**	**4/4**	0/4	0/4	**4/4**	0/4
***Salmonella***	**Enteritidis**	**O:9**	**3/3**	0/3	0/3	**3/3**	0/3
*Salmonella*	Infantis	O:7	0/1	0/1	0/1	0/1	0/1
*Salmonella*	Seftenberg	O:3	0/1	0/1	0/1	0/1	0/1
*Salmonella*	Adelaide	O:35	0/1	0/1	0/1	0/1	0/1
*Salmonella*	Wesleco	O:42	0/1	0/1	0/1	0/1	0/1
*Salmonella*	Montevideo	O:54	0/1	0/1	0/1	0/1	0/1
*Salmonella*	Tanger	O:13	0/1	0/1	0/1	0/1	0/1
*Salmonella*	Cerro	O:18	0/1	0/1	0/1	0/1	0/1
*Salmonella*	Basel	O:58	0/1	0/1	0/1	0/1	0/1
*Salmonella*	Anatum	O:3,10	0/1	0/1	0/1	0/1	0/1
*Salmonella*	Eilbeck	O:61	0/1	0/1	0/1	0/1	0/1
*Salmonella*	Worthington	O:13	0/1	0/1	0/1	0/1	0/1
*Salmonella*	Onderstepoort	O:6	0/1	0/1	0/1	0/1	0/1
*Salmonella*	Deversoir	O:45	0/1	0/1	0/1	0/1	0/1
*Salmonella*	Telaviv	O:28	0/1	0/1	0/1	0/1	0/1
*Salmonella*	Munester	O:3	0/1	0/1	0/1	0/1	0/1
*Salmonella*	Aberdeen	O:11	0/1	0/1	0/1	0/1	0/1
*Salmonella*	Inverness	O:38	0/1	0/1	0/1	0/1	0/1
*Salmonella*	Bergen	O:47	0/1	0/1	0/1	0/1	0/1
***Salmonella***	**Ruiru**	**O:21**	**1/1**	**1/1**	0/1	0/1	0/1
***Salmonella***	**Minnesota**	**O:21**	**2/2**	**2/2**	0/2	0/2	0/2
***Escherichia***	***coli***	**O:157**	**3/3**	0/3	**3/3**	0/3	0/3
*Escherichia*	*coli* (K12)		1/1*	0/1	0/1	0/1	0/1
*Escherichia*	*coli* (ECOR)		1/72*	0/72	0/72	0/72	0/72
*Shigella*	*sonnei*		1/1*	0/1	0/1	0/1	0/1
*Acinetobacter*	*baumanni*		0/1	0/1	0/1	0/1	0/1
*Klebsiella*	spp.		0/3	0/3	0/3	0/3	0/3
*Enterobacter*	*aerogenes*		0/1	0/1	0/1	0/1	0/1
*Cronobacter*	*sakazakii*		0/1	0/1	0/1	0/1	0/1
*Citrobacter*	*freundii*		0/1	0/1	0/1	0/1	0/1

*) Highly reduced infection (EOP of 10^−6^).

**Fig. 5:**
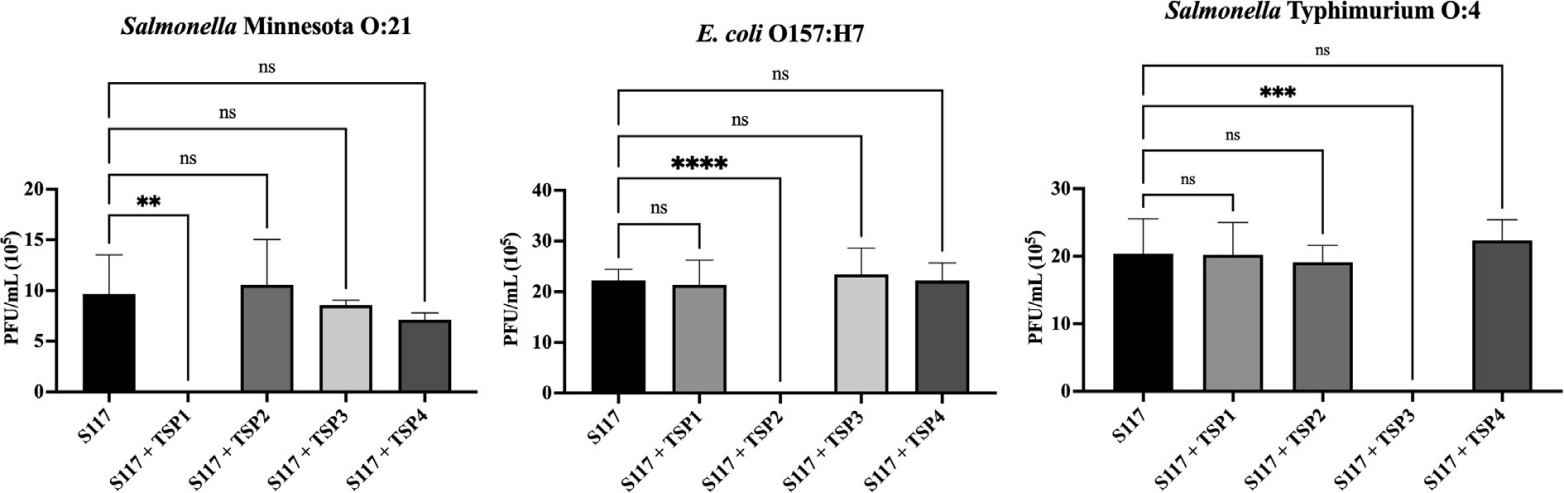
**TSPs can inhibit the infection of phage S117 of their respective hosts.** The infectivity of phage S117 in the presence of TSP1-1, TSP2-1, TSP3-1, TSP4-1 or nothing mixed with the bacterial hosts were measured using a double layered plaque assay. 0.5 mg/mL of the TSPs were able to block the infection of phage S117 on their respective hosts when a phage titer of 10^5^ PFU/mL was used. The experiment was carried out in triplicates and the error bars represent the standard deviation. The graphs were generated in prism9 where the p-values were calculated using the ordinary one-way ANOVA.

TSP3 from phage S117 belonging to subtype TSP3-1, showed the broadest host range as it bound to *Salmonella enterica* serotypes expressing O-antigens of both type O:4 and O:9. The backbone sugar residues in the O:4 and O:9 serogroups are identical (→2)-α-D-Man-(1 → 4)- α-L-Rha-(1 → 3)-α-D-Gal-(1 → ), only the branched sugars are different, where tyvelose residues are present in the O:9 group and abequose residues are found in the O:4 group on the mannose sugar residue [57]. The similarity of the O-antigens could explain why TSP3 was able form translucent clearing zones on to strains expressing both serogroups, similar to phages P22, KB1 and P27 which also recognize both O-antigens [6], [58]. However, TSP3 of S117 did not form translucent zones on all strains belonging to O:4 and O:9 serogroups (Table 4), suggesting that some strains may have modified O-antigens, like acetylation which have been implicated in phage resistance in *Salmonella* Typhimurium [59]. To validate that the O-antigen indeed is the receptor for TSP3-1, we spotted TSP3 and 10-fold serial dilutions of phage S117 on lawns of *S. Typhimurium* (O:4) mutants strains lacking known phage receptors; O-Antigen (Δ*rfbP*), flagella (Δ*flgK*), ferrichrome transporter (Δ*fhuA*) and vitamin B_12_ transporter (Δ*btuB*) (Fig. 5). As expected, TSP3 did not produce any translucent zones on the O-antigen mutant (Δ*rfbP*) and the efficiency of plating (EOP of 10^−6^) of S117 dropped significantly (Fig. 4). On the contrary, TSP3 was able to form translucent zones on the other mutants and the plaque formation of the phage was comparable to the wild type (Fig. 4), demonstrating that O-antigen of *Salmonella* indeed is the receptor for TSP3-1. Our *in silico* analysis showed that 51 phages also express TSP3s belonging to the TSP3-1 subtype (Fig. 2), thus these phages are likely to recognize O:4 and O:9 O-antigens on *Salmonella enterica* subspecies. Indeed, it has been shown that many of these phages are able to infect *Salmonella enterica* belonging to both O:4 and O:9 serogroups [30], [33], [38], [60], [61], [62]. In addition, based on the high similarity of the catalytic domains between TSP3-1 and TSP4-8 subtypes, we predict that TSPs in the TSP4-8 subtype also recognize O:4 and O:9 serogroups which have also been shown for phage SE14 [26] (Fig. 3 and Table 3).

To further validate the host recognition of TSP1, TSP2 and TSP3 of phage S117, we performed a phage inhibition assay. Here each of the TSPs were preincubated with their respective bacterial hosts prior to testing infectivity of phage S117 using a double layer plaque assay. Indeed, each of the TSPs were able to completely inhibit the infection of phage S117 (10^5^ PFU/mL) of their respectively hosts, whereas no effect was observed when the TSPs were pre-incubated with a non-host (Fig. 5). In summary, by using purified TSPs we could confirm the hosts predicted for TSP1 and TSP2 of phage S117 and identify a new host target for TSP3. Furthermore, identifying the host target of TSP3 encoded by phage S117 allowed us to predict putative hosts for 52 phages encoding TSPs in the TSP3-1 subtype. For all TSPs host recognition could be associated with specific O-antigen of the LPS.

## 4 Discussion

The *Ackermannviridae* is a novel family within the *Caudovirales* order named after the renowned Professor Hans-Wolfgang Ackermann. The family consist of the four genera; *Kuttervirus*, *Agtrevirus*, *Limestonevirus* and *Taipeivirus*, and contains phages infecting a broad range of Gram-negative bacteria including many pathogenic bacteria like *E. coli*, *Salmonella*, *Shigella* and *Klebsiella* [13], [16], [33], [37]. *Ackermannviridae* phages express a receptor-binding complex consisting of up to four TSPs recognizing different receptors on their bacterial host. For instance, the four TSPs of kuttervirus CBA120 recognize different O-antigens on *Salmonella* and *E. coli* [20]. However, only very few phages and their individual TSPs have been characterized in detail for their host range. In this study, we revealed the diversity of TSPs in the *Ackermannviridae* family through comprehensive *in silico* analysis. Based on TSP similarity we divided them into subtypes, allowing us to predict the host range of numerous uncharacterized *Ackermannviridae* phages by knowing the host recognition of a few TSPs.

Our comprehensive *in silico* analysis revealed a large number of distinct subtypes within each of the four TSPs encoded by phages belonging to the *Ackermannviridae* family. The high sequence conservation found within each subtype suggests similar receptor-biding specificity, which we validated using published data and biological experiments. On the other hand, the limited similarity at the C-terminal receptor binding modules between subtypes, suggest that each subtype may recognize different receptors. Based on the total number of subtypes each carrying diverse C-termini, potentially up to 97 different receptors may be recognized by these phages, thus displaying a vast pool of diverse RBPs for host binding within the *Ackermannviridae* family. In accordance, *Ackermannviridae* phages binds to highly variable polysaccharides on the surface of their bacterial host like the LPS or CPS [17], [20], [21], [31]. For instance, 185 and 46 O-antigens are found in *E. coli* and *Salmonella*, respectively, and 79 K-antigens are found in *Klebsiella* [5], [63], [64]. A large study of the P22-like phages (*Lederbergvirus* genus) also showed that the phages express highly diverse receptor binding modules to match the diverse O-antigens on the bacterial surface [6]. For instance, the phages HK620, P22 and Sf6 all belonging to the *Lederbergvirus* genus express structural similar TSPs, but with no sequence similarity in the receptor binding module. Each of the TSPs recognize different O-antigens found on the surface of *Salmonella*, *E. coli* and *Shigella* [2], [6]. Barbirz et al. suggested that TSPs share a common ancestor, which during the course of evolution have mutated and thereby lost sequence similarity in the receptor binding module leading to different receptor recognition, while still preserving TSP structural folds and functionality [2]. Thus, it seems likely that TSPs in the *Ackermannviridae* family have evolved to match the diverse O-antigens or K-antigens on their bacterial hosts, explaining the diversity of the TSPs in the family.

The four TSPs of kuttervirus CBA120 have previously been detailed characterized [20] and using their distinct N-terminal sequences we could assign all identified TSPs into TSP1, TSP2, TSP3 and TSP4. Further analysis of the conserved N-termini showed that the XD domains identified in TSP2 and TSP4 of CBA120 are preserved in all TSP2 and TSP4 in the *Ackermannviridae* family. These XD domains are structurally similar to the T4 Gp10 protein that facilitate interactions between proteins composing the distal tail fiber complex in phage T4 [25]. Interestingly, Gp10-like domains are not unique for *Ackermannviridae* phages, as they have been identified in many TSPs or proteins associated with TSPs, like in *E. coli* phages G7C and *Salmonella* phage SP6 [65], [66]. A recent study of TSPs in *Klebsiella* phages found multiple TSPs that contain T4 Gp10-like domains that were all located at the N-terminus [9]. As these Gp10-like domains allow complex formation hinging multiple TSPs together, they are commonly found in phages expressing multiple TSPs including phages of the *Ackermannviridae* family Thus, there may be an evolutionary relationship of Gp10-like domains with phages expressing multiple TSPs.

When comparing all TSPs, we showed that some *Kuttervirus* TSP1 and TSP3 were similar to specific TSP4s, except for the N-terminus of the TSP4. This suggests that the receptor binding modules may be exchanged between TSPs thereby alternating their host recognition. We identified a conserved sequence motif (GTTAVSL) in TSP1, TSP3 and TSP4 expressed by *Kuttervirus* phages, suggesting a site for recombination. Exchange of domains through horizontal gene transfer is a known strategy for phages to alternate their host range [36], [67]. For instance, the TSP of *Lederbergvirus* CUS-3 is similar to the C-terminus of TSP of kayfunavirus K1F [68]. Furthermore, the receptor binding module of TSP3 of kuttervirus Det7 is similar to the TSP of phage P22, proposing an exchange between even distantly related phages [21]. Thus, *Ackermannviridae* phages may alternate their host recognition by exchanging the receptor binding module between TSPs within each genus or acquiring new ones by exchanging TSPs with distant related phages thereby further expanding the vast diversity of the TSPs. Finally, the sequence similarity of the N-termini of all *Ackermannviridae* phage TSPs as well of the conserved genes flanking the TSP gene cluster provide optimal conditions for homologous recombination, allowing the entire TSP genes to be exchanged between phages thereby altering the host recognition profile. The conditions needed for exchanging whole genes or only receptor binding modules are not known, but co-infection of phages could lead to homologous recombination and thus resulting in phages acquiring new TSPs.

Host range analysis of phages are laborious when numerous species/strains must be tested, especially for phages expressing multiple RBPs and is limited by the strains available in the laboratory. Here, we determined host recognition of multiple TSPs by knowing the host recognition of only a few TSPs using our *in silico* analysis and published data of host ranges. In addition, we purified the TSPs of *Kuttervirus* S117 and spotted these on our large *Salmonella* and *E. coli* collection, allowing us to validate our predictions as well as to identify the host for TSPs of the TSP3-1 subtype. Other studies have also used computational tools to predict phage host specificity [69], [70], [71], [72]. For instance, the bioinformatics tool HostPhinder predicts the phage-host interactions by comparing the genomic similarities through *k*-mers by the querying phage and an established database of phages with known hosts [70]. Also, a recent paper used machine-learning to predict the host range based on the RBP sequences of phages [72]. The authors extracted RBP sequences of phages known to infect ESKAPE organisms as well as *E. coli*, *Salmonella enterica* and *Clostridium diffcile* species and assign the host recognition of phages based on the sequence similarity in the RBPs. Their method allowed them to assign the host recognition of RBP down to the species level. However, Boeckaerts et al. also mentioned that their approach was not able to distinguish the RBP recognition in phages expressing multiple RBPs [72]. Our approach allowed us to identify host recognition of individual TSPs of a phage expressing multiple RBPs and predict the host range of the phage as well as the receptor recognized by each TSP. Since our approach is restricted by the species/strains available in a given lab, the host and receptors of numerous TSPs of *Ackermannviridae* are still to be identified. In summary, our work provides novel insight to the high diversity of TSPs in the *Ackermannviridae* and showed that comprehensive *in silico* analysis is an ideal tool for predicting host interaction in multiple phages belonging to the same family, when combined with biological data demonstrating host recognition. Future identification of the host recognition of individual TSPs in *Ackermannviridae* phages will allow optimization of host prediction tools and provide insight into the evolution of TSPs in the *Caudovirales* order.

## CRediT authorship contribution statement

**Anders Nørgaard Sørensen:** Conceptualization, Methodology, Validation, Formal analysis, Investigation, Writing – original draft, Writing – review & editing. **Cedric Woudstra:** Investigation, Writing – review & editing. **Martine C. Holst Sørensen:** Conceptualization, Visualization, Writing – review & editing, Funding acquisition. **Lone Brøndsted:** Conceptualization, Project administration, Supervision, Visualization, Writing –review & editing, Project administration, Funding acquisition.

## Declaration of Competing Interest

The authors declare that they have no known competing financial interests or personal relationships that could have appeared to influence the work reported in this paper.
