# Supplementary material for: Subtypes of tail spike proteins predicts the host range of Ackermannviridae phages
Source: Comput Struct Biotechnol J. 2021 Aug 21;19:4854–67. doi: 10.1016/j.csbj.2021.08.030 (PMC8432352; doi:10.1016/j.csbj.2021.08.030)
Supplement: Supplementary data 1 [file mmc1.docx]

# Supplementary data

Table S1: Primers used for cloning TSPs into the pET-28a(+) vector

| **primers** | **Sequence** |
| --- | --- |
| pET28-F  pET28-R | 5’- CTCGAATTCGGATCCGCG-3’  5’- CTCCGTCGACAAGCTTGC-3’ |
| TSP1-F  TSP1-R | 5’-GCAAGCTTGTCGACGGAGCTATGATCCTATAATTTTATAGGTTAGAGAAGTATTACC-3’  5’-CGCGGATCCGAATTCGAGATGAACGAAATGTTTAGTCAAGGTG-3’ |
| TSP2-F  TSP2-R | 5’-GCAAGCTTGTCGACGGAGTTAAATAGAAGAATCCAATATACGGTAACTAATTAAC-3’  5’-CGCGGATCCGAATTCGAGATGACCAGAAATGTAGAAGAATTATTCGG-3’ |
| TSP3-F  TSP3-R | 5’- GCAAGCTTGTCGACGGAGTTAAAGTGTGTATGTTACATAACTTCCATC -3’  5’- CGCGGATCCGAATTCGAGATGATTTCTCAATTCAATCAACCACG -3’ |
| TSP4-F  TSP4-R | 5’- GCAAGCTTGTCGACGGAGCTATGATGTAAATTTCATTGCATCCCATG -3’  5’- CGCGGATCCGAATTCGAGATGGCCAACAAACCAACACAG -3’ |


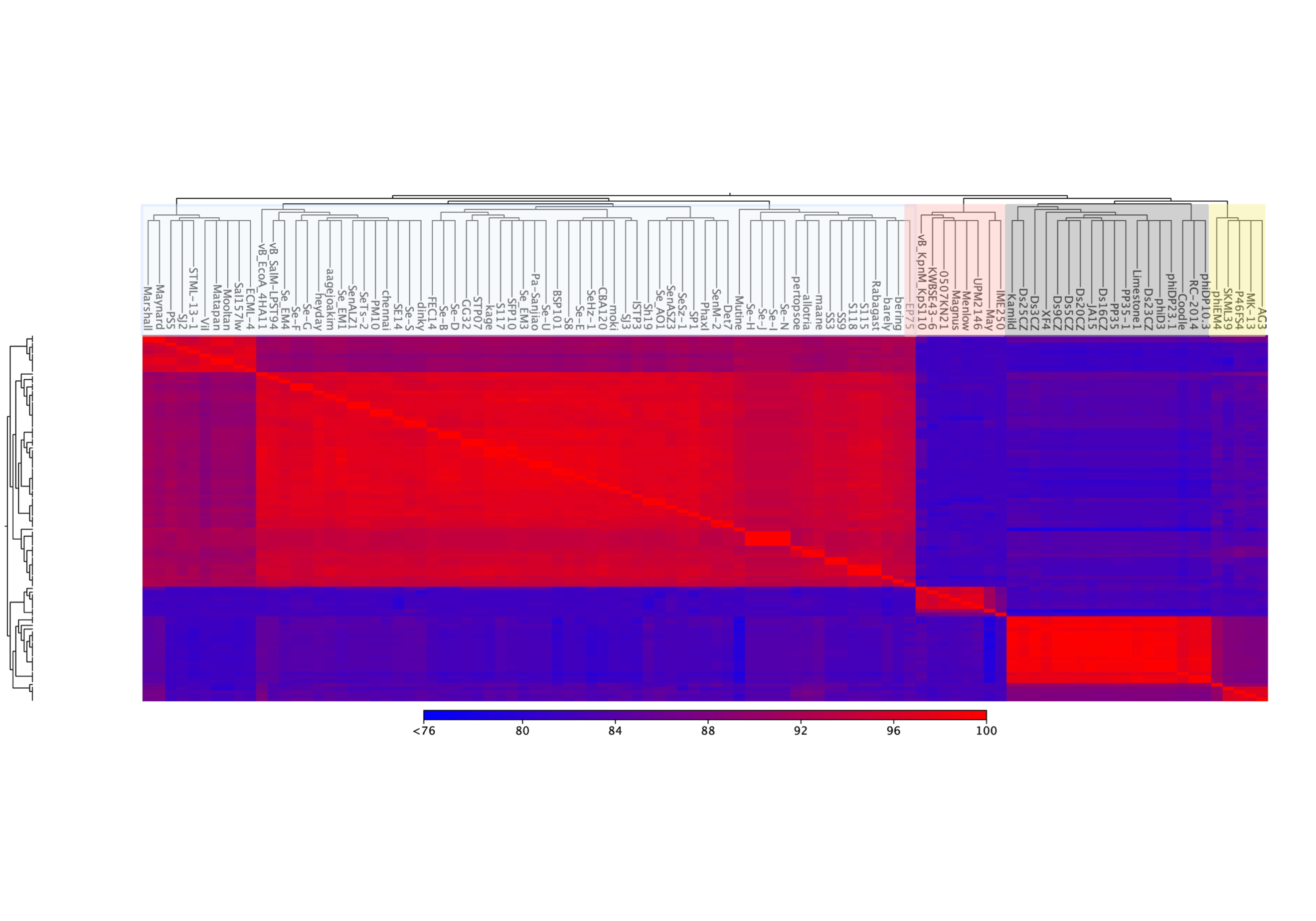


**Figure S1: The genomes of the *Limestonevirus* phages are more similar compared to the other phage genera.** The average nucleotide identity was calculated and visualized in a heat map (Blue: *Kuttervirus*, red: *Taipeivirus*, gray: *Limestonevirus*: gray and *Agtrevirus*: yellow). The analysis was done in CLC Workbench 21 software

#

**Figure S2: The structural domains of the TSP can be swapped between TSPs.** A) Alignment of TSPs in the TSP3-3 and TSP4-2 subtypes. B) TSP3-1 subtypes were aligned against TSP4-8. Common for all alignments are the conservation started at the same amino acid position 65 and 391 for TSP1s and TSP3s, and TSP4s, respectively. Multiple alignment was done in CLC Main Workbench software.


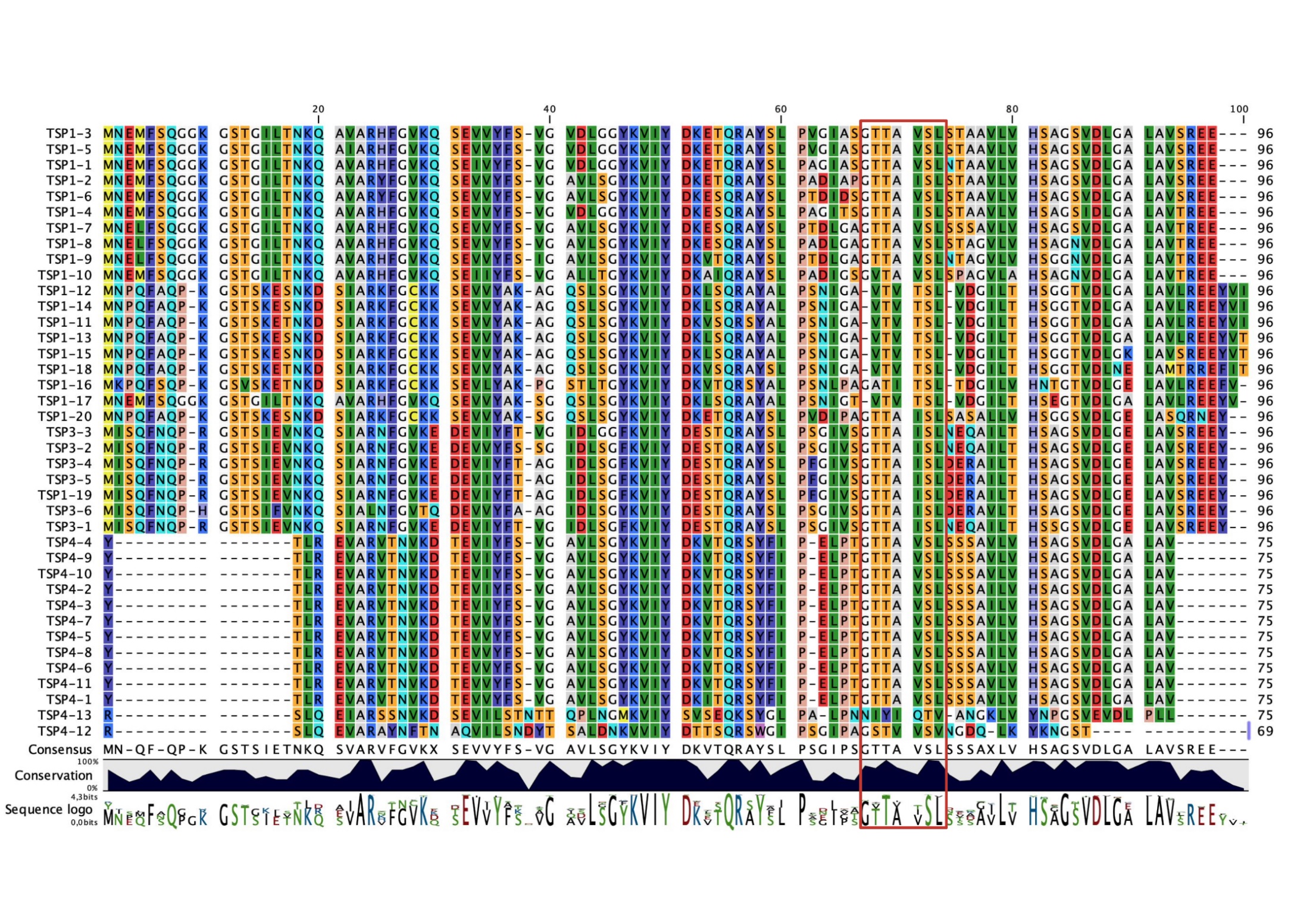


**Figure S3: The TD1 domain are conserved in the TSP1, TSP3 and TSP4 subtypes expressed in the *Kuttervirus* genus.** The sequence spanning the TD1 domain (residues 1-96) of *Kuttervirus* TSP1 and TSP3 subtypes were aligned with the sequence spanning the TD1 domain (residues 344-421) of *Kuttervirus* TSP4 subtypes. Red box: place of recombination in TSP1-1 and TSP4-7 subtypes, TSP3-1 and TSP4-8, and TSP3-3 and TSP4-2 subtypes.

**Figure S4: The TSPs are resistant to SDS**. Purified TSPs were subjected to SDS-PAGE boiled (D) or un-boiled (N) to examine if they were resistant to SDS. **Asterisk**: TSP trimer. L: protein ladder.
